# The Peptide-Directed Lysosomal Degradation of CDK5 Exerts Therapeutic Effects against Stroke

**DOI:** 10.14336/AD.2018.1225

**Published:** 2019-10-01

**Authors:** Ya-Fan Zhou, Jing Wang, Man-Fei Deng, Bin Chi, Na Wei, Jian-Guo Chen, Dan Liu, Xiaoping Yin, Youming Lu, Ling-Qiang Zhu

**Affiliations:** ^1^Department of Pathophysiology, School of Basic Medicine, Tongji Medical College, Huazhong University of Science and Technology, Wuhan, China; ^2^Department of Radiology, Union Hospital, Tongji Medical College, Huazhong University of Science and Technology, Wuhan, Hubei, China; ^3^Department of Pathology, The First Affiliated Hospital of Zhengzhou University, Department of Pathology, School of Basic Medicine, Zhengzhou University, Zhengzhou, China; ^4^Department of Pharmacology, School of Basic Medicine, Tongji Medical College, Huazhong University of Science and Technology, Wuhan, China; ^5^The Institute for Brain Research, Collaborative Innovation Center for Brain Science, Huazhong University of Science and Technology, Wuhan, China.; ^6^Department of Neurology, The Affiliated Hospital of Jiujiang University, Jiujiang, Jiangxi, China

**Keywords:** Stroke, CDK5, peptide-directed degradation

## Abstract

The aberrant activation of CDK5 has been implicated in neuronal death in stroke. The goal of this study is to determine whether knocking down CDK5 by a peptide-directed lysosomal degradation approach is therapeutically effective against stroke. We synthesized a membrane-permeable peptide that specifically binds to CDK5 with a chaperone-mediated autophagy targeting motif (Tat-CDK5-CTM) and tested its therapeutic effects on a mouse model of ischemic stroke. Our results showed that Tat-CDK5-CTM blocked the CDK5-NR2B interaction, resulting in the degradation of CDK5, which in turn prevented calcium overload and neuronal death in cultured neurons. Tat-CDK5-CTM also reduced the infarction area and neuronal loss and improved the neurological functions in MCAO (Middle cerebral artery occlusion) mice. The peptide-directed lysosomal degradation of CDK5 is a promising therapeutic intervention for stroke.

Ischemic stroke is a devastating and major cause of morbidity and mortality worldwide. However, due to the narrow time window of thrombolytic therapy, new pharmacological therapeutic approaches are still necessary. Cyclin-dependent kinase 5 (CDK5) is a proline-directed serine/threonine kinase that interacts with NR2B and phosphorylates NR2B to promote ischemic neuronal death [1],[2]. Targeting aberrant CDK5 is neuroprotective for the neuronal loss, tauopathy, and microglial hyperreactivity induced by stroke [1, 3, 4]. Previously, a membrane-permeant targeting peptide-based method that rapidly and reversibly knocks down endogenous proteins through chaperone-mediated autophagy (CMA) had been validated [5]. In this study, we synthesized a membrane-permeable peptide (Tat-CDK5-CTM) that specifically disrupts the binding of CDK5 and NR2B and then leads to the degradation of CDK5 by a lysosome-mediated pathway. We found that the administration of Tat-CDK5-CTM not only retards calcium overload and neuronal death in oxygen and glucose deprivation (OGD)-treated neurons but also rescues neuronal loss and the infarction area and recovers motor coordination and memory retention.

**Figure 1.**
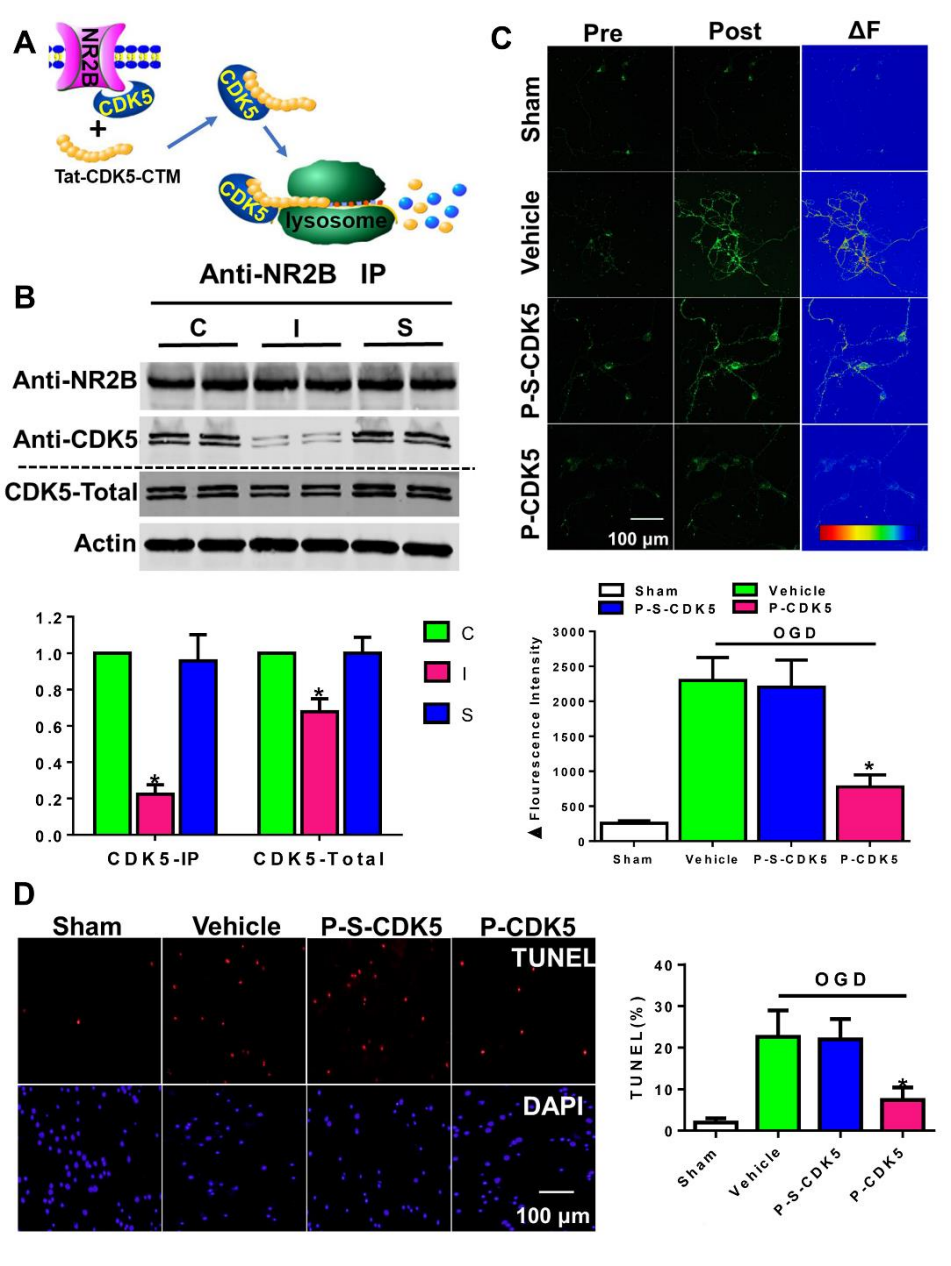
Tat-CDK5-CTM Protects Against OGD-induced neuronal injury. (A) Tat-CDK5-CTM disrupts the binding of NR2B and CDK5 and leads to the degradation of CDK5. (B) Tat-CDK5-CTM inhibits the NR2B-CDK5 interaction (upper panel) and promotes CDK5 degradation (lower panel) in cultured cortical neurons. C/I/S, OGD neurons treated with vehicle/Tat-CDK5-CTM/Tat-s-CDK5-CTM. Quantitative analysis of the Western blot results. Data are presented as the means ± SEM (ANOVA, *P<0.05; n=5). (C) Fluo3Am Ca^2+^ images were taken before (Pre) and after (Post) OGD treatment. The ΔF were calculated. Data are presented as the means ± SEM (ANOVA, *P<0.05; n=6). (D) Representative images of TUNEL staining. Vehicle, OGD neurons treated with vehicle. P-S-CDK5, OGD neurons treated with Tat-s-CDK5-CTM, P-CDK5, OGD neurons treated with Tat-CDK5-CTM. Quantitative analysis of the TUNEL staining results. Data are presented as the means ± SEM (ANOVA, *P<0.05; *vs* vehicle group without specific explanation, n=8).

## MATERIALS AND METHODS

### Animals

Adult (90 ± 5 days old) male C57BL/6J mice were used in all experiments. The mice were housed individually under standard conditions of temperature and humidity and a 12 h light/dark cycle (lights on at 08:00) with food and water *ad libitum*. All experiments were carried out according the Institutional Guidelines of the Animal Care and Use Committee (Huazhong University of Science and Technology).

**Figure 2.**
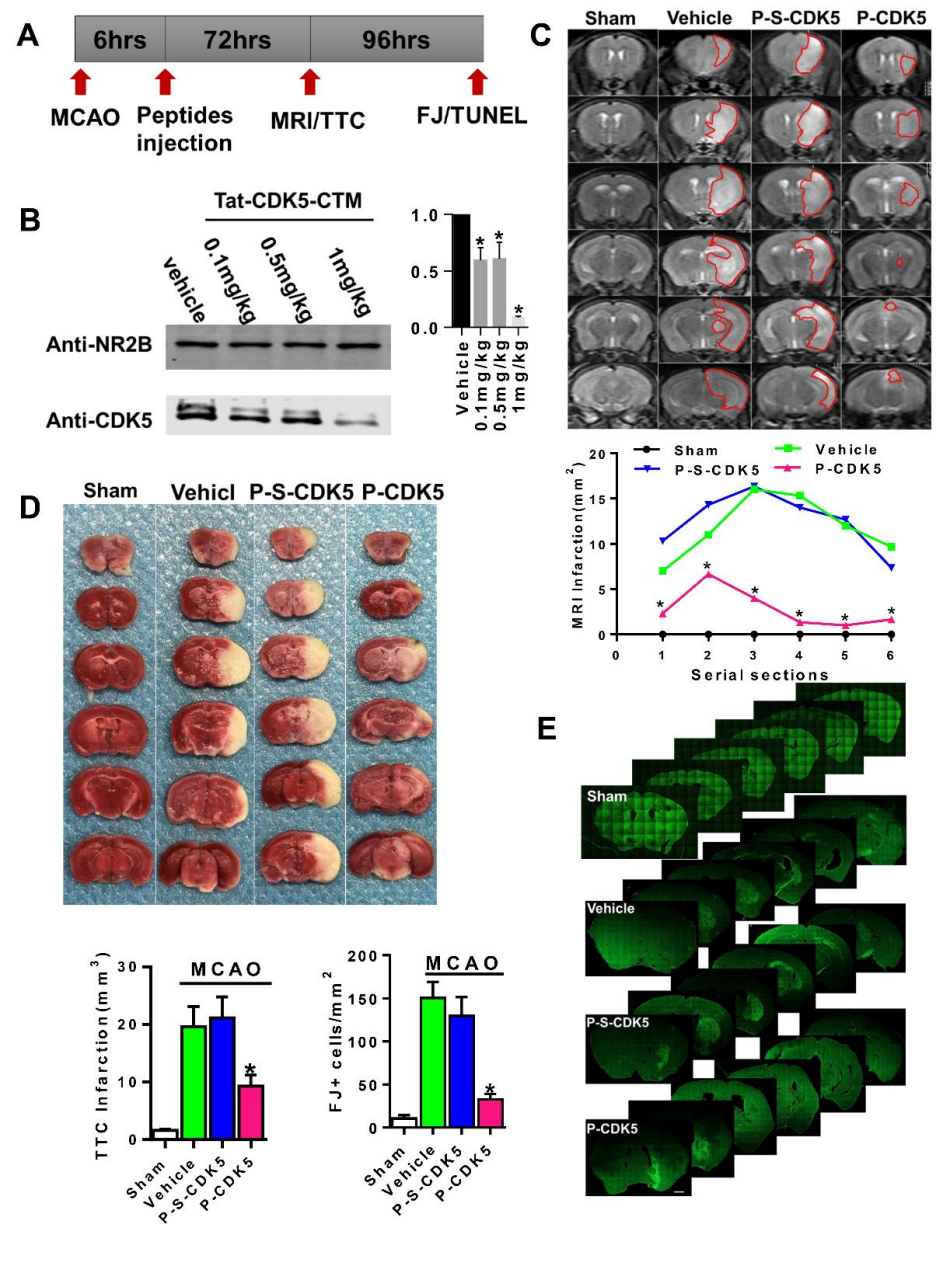
Administration of Tat-CDK5-CTM protects against stroke damage *in vivo*. (A) A diagram of the experimental schedule. (B) The effect of different doses of Tat-CDK5-CTM on the CDK5 protein level. Quantitative analysis of panel B. (ANOVA, *P<0.05; n=6). (C) MRI images. Vehicle, mice treated with vehicle and MCAO. P-S-CDK5, mice treated with Tat-s-CDK5-CTM and MCAO, P-CDK5, mice treated with Tat-CDK5-CTM and MCAO. Quantitative analysis of the areas of cerebral infarct in different serial sections in panel C. Data are presented as the means ± SEM (ANOVA, *P<0.05; n=7). (D) Representative images of TTC staining. A graph shows the sizes of the cerebral infract. Data are presented as the means ± SEM (ANOVA, *P<0.05; n=7). (E) Representative images show the fluoro-jade C(FJ)-labeled cells in the cortex and striatum of mice. The scale bar in serial pictures is 2.5 mm. The bar graph shows the numbers of FJ-labeled cells. Data are presented as the means ± SEM (ANOVA, *P<0.05; n=9).

### Peptides

Tat-CDK5-CTM (YGRKKRRQRRR-RRPPRSPDHKRYFRDKE-KFERQKILDQRFFE) or Tat-scramble-CDK5 (YGRKKRRQRRR-PHPRSRPRKEDDKRYFR-KFERQKILDQRFFE) peptides with 99.18% purity were synthesized by ChinaPeptides Co., Ltd. (Jiangsu, China). The peptides were numbered, and the investigators did not know which peptide was applied in the experiments.

### Cell Death Analysis

Twenty-four hours after oxygen/glucose deprivation (OGD) and peptide administration, cultured neurons were stained with TUNEL and PI. The number of TUNEL- and PI-labeled cells were expressed as a percentage of the total numbers of DAPI-labeled cells per condition. Seven days after MCAO and peptide administration, the brain sections were stained with fluoro-jade C (FJ) and TUNEL. The number of FJ- and TUNEL-positive cells in each group was counted.

### Behavioral Analysis

Neurological performance was scored daily using modified 7-point neurological scales. A rotarod treadmill was used to measure the motor coordination. The behavioral analysis was always assessed by blinded independent investigators who were unaware of the experimental conditions and treatments.

### Data Analysis

All variable values in the text and figure legends are presented as the means ± SEM. Western blot results, infarct volumes, TUNEL- or PI- or FJ-positive cells, and neurological scores were analyzed using ANOVA with Student-Newman-Keuls (SNK) tests. The behavioral test data were analyzed by two-way repeated-measures ANOVA with Bonferroni comparisons. Significant differences were defined as P <0.05.

## RESULTS

### Tat-CDK5-CTM Intercepts the NR2B-CDK5 Interaction

The cortical neurons were treated with OGD for 60 min and then the neurons were incubated for 2 h with Tat-CDK5-CTM (Fig. 1A) or the scrambled peptide at a concentration of 5 μM. As expected, Tat-CDK5-CTM effectively disrupted the binding of NR2B and CDK5 and led to the degradation of CDK5 (Fig. 1B) via a lysosome-mediated pathway (Supplementary Fig. 1A-B).

### Tat-CDK5-CTM Protects Against OGD-induced neuronal injury

By using Fluo3Am Ca^2+^ imaging, we found that Tat-CDK5-CTM significantly reduced calcium influx during the OGD insult (Fig. 1C). We also examined neuronal death by TUNEL and PI staining and found that Tat-CDK5-CTM reduced the percentage of TUNEL- or PI-positive cells when compared with vehicle- or Tat-s-CDK5-CTM-treated OGD neurons (Fig. 1D and Supplementary Fig. 2A-B), suggesting that Tat-CDK5-CTM can protect against stroke damage *in vitro*.

### Tat-CDK5-CTM Protects Against Stroke in vivo

Adult male mice underwent MCAO for 60 min followed by reperfusion, and all animals were confirmed *via* monitoring cerebral blood flow. Six hours after the MCAO operation, the animals were injected (intravenously) with the vehicle, Tat-CDK5-CTM or Tat-s-CDK5-CTM at a single dose of 0.1, 0.5, or 1 mg/kg body weight (Fig. 2A). We found that Tat-CDK5-CTM effectively distributed to the whole brain (Supplementary Fig. 3A-B), and at a dose of 1 mg/kg, Tat-CDK5-CTM sufficiently uncoupled CDK5 protein from the NR2B complex in the cortical neurons of MCAO mice (Fig. 2B).

The mice were subjected to MRI at 72 h after the peptide injection. The MRI results showed that the Tat-CDK5-CTM treatment effectively decreased the area of cerebral infarction in all slices (Fig. 2C). After MRI, the mice were sacrificed for analysis with 2,3,5-triphenyltetrazolium chloride (TTC) staining. We found that Tat-CDK5-CTM at a single dose of 1 mg/kg decreased the cerebral infarction from 19.6±2.3 mm^3^ (vehicle) and 20.7±2.6 mm^3^ (Tat-s-CDK5-CTM) to 9.7±1.2 mm^3^ (Fig. 2D).

Cell death in the mouse brain sections was detected at 7 days after ischemic insult and peptide administration by FJ staining. In this study, the mice were treated with Tat-CDK5-CTM/ Tat-s-CDK5-CTM/vehicle at 6 hours after MCAO operation. We found that Tat-CDK5-CTM reduced the number of FJ-labeled cells in the vulnerable brain regions. The magnitude of FJ labeling reduction was similar to the reduction of the brain infarction (Fig. 2E). Similar rescue effects were also detected by TUNEL staining (Supplementary Fig. 3C-D). Thus, Tat-CDK5-CTM can protect against stroke damage *in vivo*.

### Tat-CDK5-CTM improves neurological function

**Figure 3.**
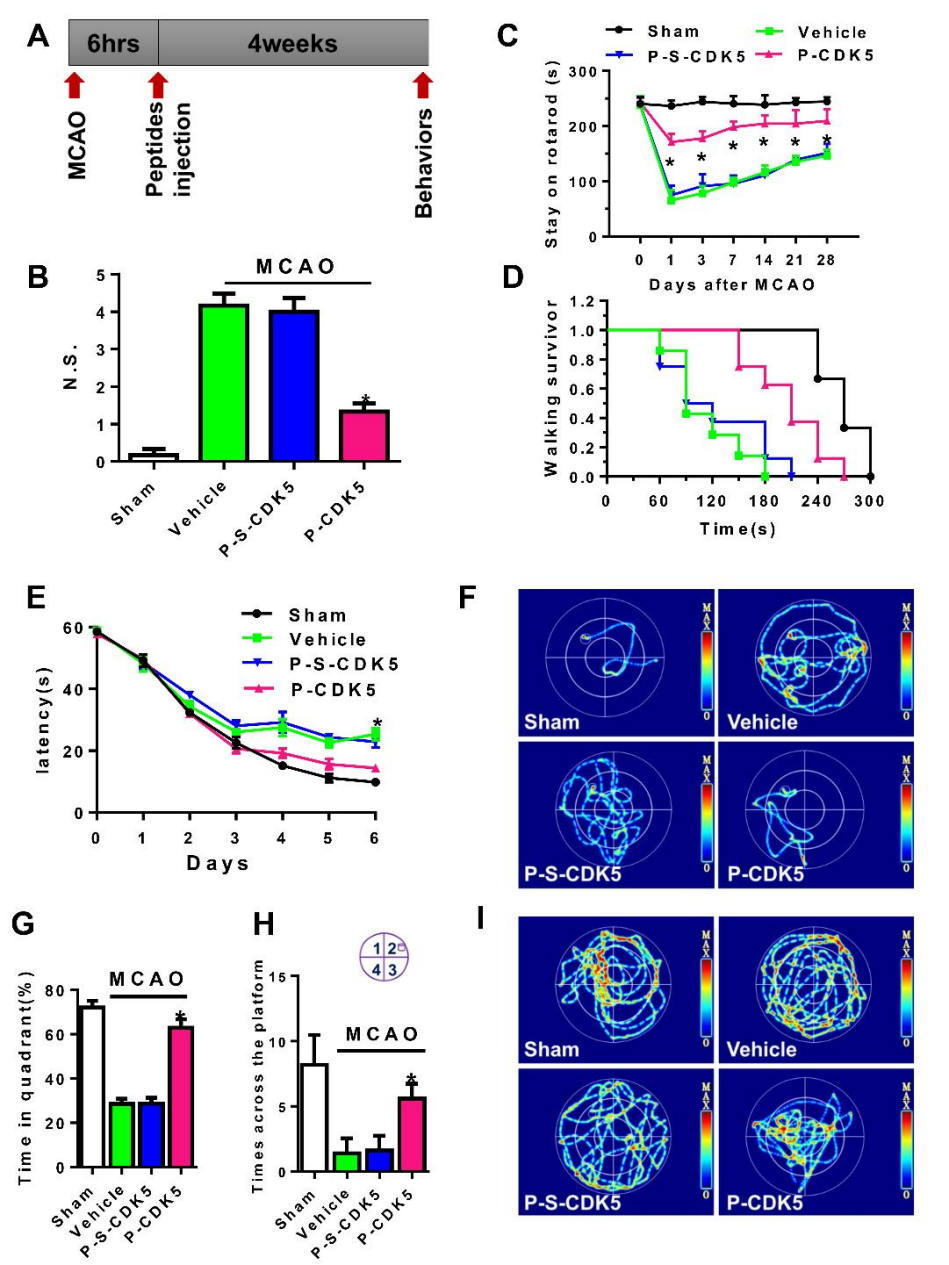
Tat-CDK5-CTM improves overall neurological functions. (A) Adult mice were administered (intravenously) a single dose (1 mg/kg) of vehicle, Tat-CDK5-CTM, or Tat-s-CDK5-CTM at 6 hours after operation with sham or middle cerebral artery occlusion (MCAO). (B) Overall neurological scores (N.S.) (ANOVA, *P<0.05; n=11). (C-D) The performance on rotarod (C) and walking survivor (D) (ANOVA, *P<0.05; n=11). (E) The latency to reach a hidden platform on days 1-6 (ANOVA, *P<0.05; n=11). (F) The representative traces on day 7. (G) A bar graph shows the percentage of time spent in the target quadrant during the probe trial (ANOVA, *P<0.05; n=11). (H) The total times crossing the platform region in the probe trial (ANOVA, *P<0.05; n=11). (I) The representative traces on day 9 for the probe trial.

We then performed behavioral tests at 4 weeks after MCAO operation and peptide injection (Fig. 3A). The mice treated with 1 mg/kg body weight of Tat-CDK5-CTM displayed a better neurological score (Fig. 3B). In the rotarod treadmill test, Tat-CDK5-CTM treatment significantly elevated the staying time on the rotarod and increased the walking survivors (Fig. 3C-D), suggesting better motor coordination. In the Morris Water Maze Tests and Reversal Maze Tests, Tat-CDK5-CTM treatment reduced the latency and increased the duration times in the target quadrant and the crossing times to the platform (Fig. 3E-I). These data strongly suggest that Tat-CDK5-CTM treatment improves neurological function in MCAO mice.

## DISCUSSION

Here, we demonstrate that the application of the Tat-CDK5-CTM peptide, which can effectively disrupt the binding of CDK5 with NR2B and lead to the lysosomal-mediated degradation of CDK5, is protective against stroke damage both *in vitro* and *in vivo*. In MCAO animals, the increased expression of CDK5 and p35 was first detected in periinfarcted rat neurons [6]. CDK5 has been demonstrated to phosphorylate NMDA receptors, which in turn results in calcium overload and excitotoxicity, thereby inducing cell death following ischemia in hippocampal neurons [7]. Tat-CDK5-CTM not only disrupts the binding of CDK5 with NR2B to block excitotoxicity but also promotes the degradation of CDK5, which will further diminish the potential phosphorylation of other downstream targets of CDK5[8]. Indeed, our study provided experimental evidence for the protective effects of Tat-CDK5-CTM on neuronal death and neurological function.

## Supplementary Materials

The Supplemenantry data can be found online at: www.aginganddisease.org/EN/10.14336/AD.2018.1225
