# Supplementary material for: The Peptide-Directed Lysosomal Degradation of CDK5 Exerts Therapeutic Effects against Stroke
Source: Aging Dis. 2019 Oct 1;10(5):1140–5. doi: 10.14336/AD.2018.1225 (PMC6764726; doi:10.14336/AD.2018.1225)
Supplement: Supplementary file 1 — The Supplemenantry data can be found online at: www.aginganddisease.org/EN/10.14336/AD.2018.1225 [file AD-10-5-1140-s.pdf]

SUPPLEMENTARY DATA

The Peptide-Directed Lysosomal Degradation of CDK5 Exerts Therapeutic Effects against Stroke

Ya-Fan Zhou[1,#], Jing Wang[2,#], Man-Fei Deng[1], Bin Chi[2], Na Wei[3], Jian-Guo Chen[4,5], Dan Liu[1,5], Xiaoping Yin[6], Youming Lu[5], Ling-Qiang Zhu[1,5]

[1]Department of Pathophysiology, School of Basic Medicine, Tongji Medical College, Huazhong University of Science and Technology, Wuhan, China
[2]Department of Radiology, Union Hospital, Tongji Medical College,Huazhong University of Science and Technology, Wuhan, Hubei, China
[3]Department of Pathology, The First Affiliated Hospital of Zhengzhou University, Department of Pathology, School of Basic Medicine, Zhengzhou University, Zhengzhou, China
[4]Department of Pharmacology, School of Basic Medicine, Tongji Medical College, Huazhong University of Science and Technology, Wuhan, China
[5]The Institute for Brain Research, Collaborative Innovation Center for Brain Science, Huazhong University of Science and Technology, Wuhan, China.
[6]Department of Neurology, The Affiliated Hospital of Jiujiang University, Jiujiang, Jiangxi, China

© 2018. Zhou YF et al. Published online at http://www.aginganddisease.org/EN/10.14336/AD.2018.1225

SUPPLEMENTARY DATA

SUPPLEMENTARY MATERIALS AND METHODS

Primary neuron culture

The cerebral cortex was isolated from the C57BL/6J E20 embryos. Cells were dissociated and purified as we described previously[1] and plated at a density of 150cells/mm^2 on 20mm coverslips coated with 10ug/ml poly-D-lysine and 5ug/ml lamina. The cells were cultured in DMEM-F12 Medium (Invitrogen) with 10% FBS (Gibco), and the medium was replaced by serum-free Neurobasal Medium (Invitrogen) plus 2% B27 (Gibco) 4h later. The medium was half replaced every 3 days. Some coverslips were stained for Tuj1, a neuronal marker to confirm that the cells were >85% neurons. The cultures were used for OGD, Western blotting, Fluo3Am Ca^{2+} imaging, TUNEL staining and PI staining. The number of TUNEL-labeled cells and PI-labeled cells were expressed as a percentage of total numbers of DAPI-labeled cells per condition.

Oxygen/Glucose Deprivation (OGD)

The cultured neurons were washed 3 times with 500ul of deoxygenated glucose-free bicarbonate solution, and then transferred to an anaerobic chamber containing 5% CO2, 10% H2 and 85%N_2 at 37°C for 60 min. After that, the neurons were washed with normal culturing medium and maintained for 72h at 37°C in a 5% CO2, 10% H2 and 85%O_2 atmosphere.

Fluo3Am Ca^{2+} Imaging

Neurons at 8 DIV were loaded with 10 uM Fluo3Am (Invitrogen) in HBSS (Invitrogen) for 30 min at 37°C followed by a 30 min wash[2]. Images were acquired before and after OGD treatment.

Focal Cerebral Ischemia

Middle cerebral artery occlusion (MCAO) surgery to replicate the focal cerebral ischemia model described previously[3]. Briefly, a 7/0 surgical nylon monofilament with rounded tip was introduced into the left internal carotid through the external carotid stump and advanced 10–13 mm past the carotid bifurcation. The filament was left in place for 60 min and then withdrawn. The sham-operated animals were treated identically, except that the middle cerebral artery was not occluded after the neck incision.

Peptides Dose–Effect Experiments

Mice underwent a 60-minute MCAO were injected (intravenously) with vehicle (control) /Tat-CDK5-CTM /Tat-s-CDK5 at a single dose of 0.1, 0.5, 1, or 2 mg/kg body weight. Twelve hours later, all mice were sacrificed, and the cortical protein were harvested. The proteins were precipitated with anti-NR2B and blotted with anti-NR2B and anti-CDK5 antibodies, respectively. The ratio of the precipitated NR2B in each treatment was nonsignificant different. The dose of 1mg/kg was chosen to be used in the next experiments.

Magnetic Resonance Imaging

The animals were kept in an environment with the temperature was 24 °C and the humidity was 50%. MRI scan were performed using a 3 Tesla scanning system (GE Discovery MR750; GE Healthcare, USA）. A small animal receive coil (Chenguang, shanghai, Made in China) was used to maximize image resolution and quality. T2-weighted Coronal sections were as follow: fast spin echo sequence with repetition time (TR) of 4260 ms and echo time (TE) of 97 ms; matrix 256; field of view (FOV) 7.0 ∗ 1.0 cm; slice thickness was 4 mm; interslice gap 1.5 mm.

TTC Staining

© 2018. Zhou YF et al. Published online at http://www.aginganddisease.org/EN/10.14336/AD.2018.1225

SUPPLEMENTARY DATA

Mice were sacrificed 3 days after ischemic reperfusion, and brains were frozen at −20°C for 15 min. then brains were cut into 7 slices from the frontal tips at 1mm, and sections were incubated in 2% TTC at 37°C for 15 min[3]. The infarctions were determined by the areas that did not stain with TTC.

FJ-C Staining and TUNEL Staining

Animals were deeply anesthetized 7days after ischemia and perfused with ice-cold 0.9% saline followed by ice-cold 4% paraformaldehyde in 0.1 phosphate-buffered saline (PBS); Brains were cut into 20um slices and put on a slide glass. the sections were dried overnight. FLUORO-JADE C (EMD Millipore) and TUNEL staining kit (TUNEL Apoptosis Assay Kit, Roach) was used to stain death cells in 20-μm coronal frozen sections[4]. Pictures of labeled sections were taken for 4 adjacent fields (beginning at a random starting position) with a confocal laser-scanning microscope (LSM710, Zeiss). All labeled cells were counted using Image J.

Immunofluorescence staining

Cultured cells were fixed by ice-cold 4% paraformaldehyde in 0.1 phosphate-buffered saline (PBS). Rabbit or Mouse serum was used for blocking for 1h. Next the antibody to LAMP-1(Abcam, ab25630,1:1000) and Tat (Abcam, ab43014, 1:1000) were incubated overnight, and then 2h of incubation with anti-mouse Alexa 546 and anti-rabbit Alexa 488, as well as 5 min incubation with 4′,6-diamidin-2-phenylindol (DAPI, 1:10 000). To visualize uptake of the peptide (Tat-CDK5-CTM) in brain, free-floating frozen sections (30 μm) were incubated at 4°C with Rabbit anti-Tat antibody (Abcam, ab43014, 1:1000) for 48h and then 2h of incubation with anti-Rabbi Alexa488. Pictures were taken with a confocal laser-scanning microscope (LSM710, Zeiss).

Neurological Score

Neurological performance was scored 28 days after ischemia using Longa 5-point neurological scales; 0, no observable neurological deficits; 1, unable to extend the offside forelimb; 2, decreased grip of the offside forelimb; 3, mild circling to the offside; 4, spontaneous offside circling; and 5, falling to the contralateral side. Score were ranked from 0 to 5. Neurological performance was evaluated by the blinded independent investigators.

Rotarod Treadmill Test

Rotarod treadmill for mice was used to measure the motor coordination of the animals after operation and treatment[5]. The rotarod was under the accelerating rotor mode (10 speeds from 4 to 40 rpm for 5 min). The retention time was from the animal mounted the rod to it fell off, and the mice was recorded as the walking survivor if it walked for 300s on the accelerating rotating rod. All mice were trained 3 trials per day for 2 days before surgery, and the mean retention time was recorded to obtain stable baseline values. Mice underwent Rotarod Treadmill Test 3 times a day in the following 4 weeks after ischemic insult and peptide administration.

SUPPLEMENTARY DATA

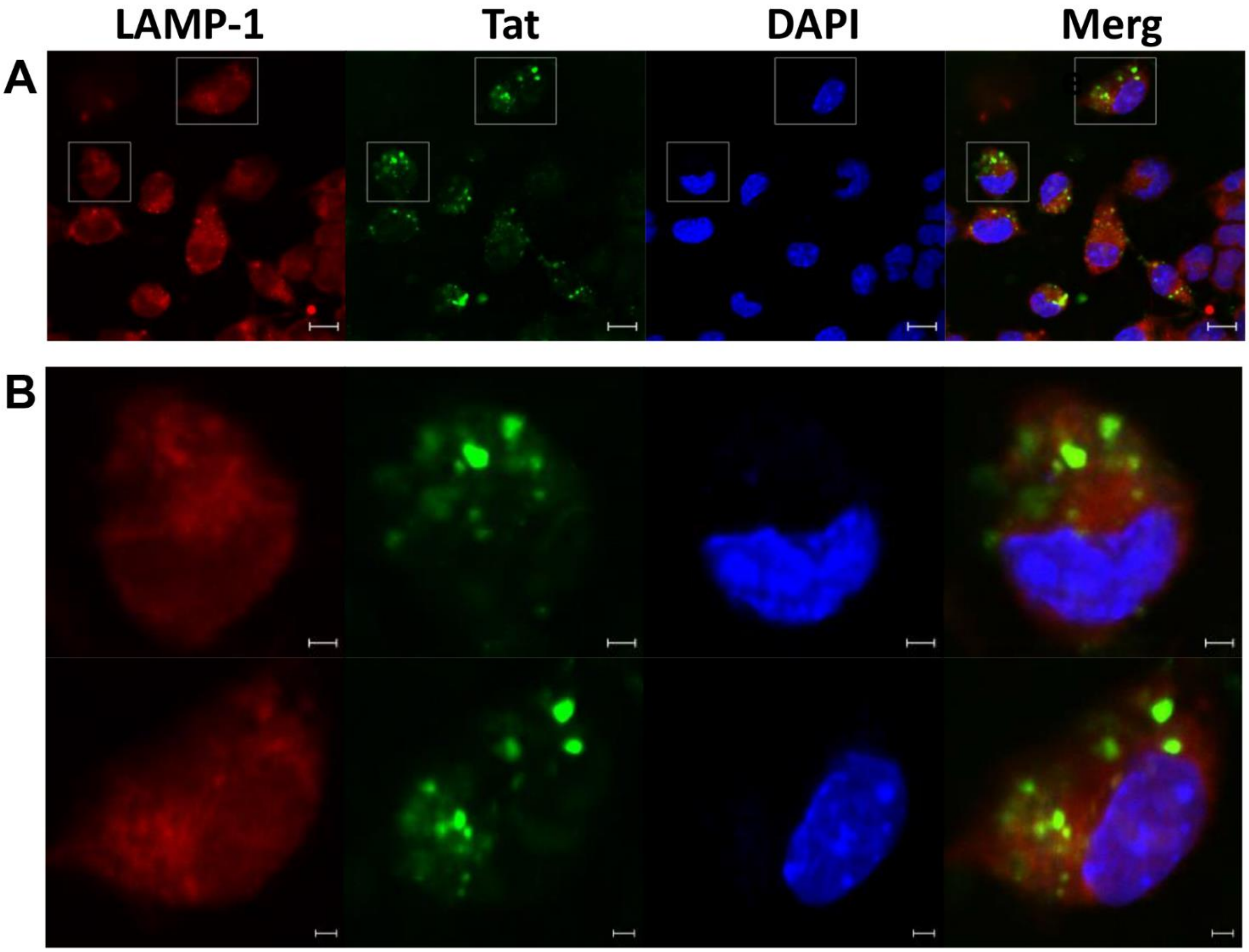

Supplement Figure 1. Tat-CDK5-CTM promotes CDK5 degradation through lysosomal involved pathway. (A) Representative images of immunofluorescence staining of cultured N2a cells shows Tat-CDK5-CTM promotes Cdk5 degradation through lysosomal involved pathway. Bar=10μm. **(B)** Representative enlarged images of immunofluorescence staining of cultured N2a cells shows Tat-CDK5-CTM promotes Cdk5 degradation through lysosomal involved pathway. Bar=2μm

.

© 2018. Zhou YF et al. Published online at http://www.aginganddisease.org/EN/10.14336/AD.2018.1225

SUPPLEMENTARY DATA

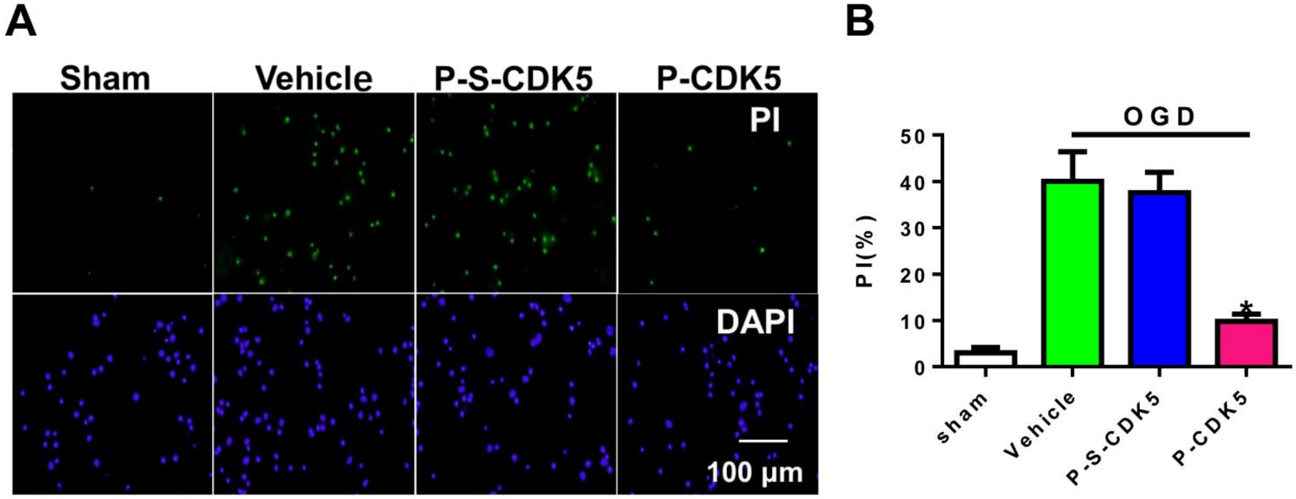

Supplement Figure 2. Tat-CDK5-CTM protects against OGD-induced neuronal injury. (A) Representative images of PI staining. Vehicle, neurons treated with vehicle and OGD. P-S-CDK5, neurons treated with Tat-s-CDK5-CTM and OGD, P-CDK5, neurons treated with Tat-CDK5-CTM and OGD. **(B)** The quantitative analysis of the PI staining. Data are mean ± SEM (ANOVA, *P<0.05; n=8).

© 2018. Zhou YF et al. Published online at http://www.aginganddisease.org/EN/10.14336/AD.2018.1225

SUPPLEMENTARY DATA

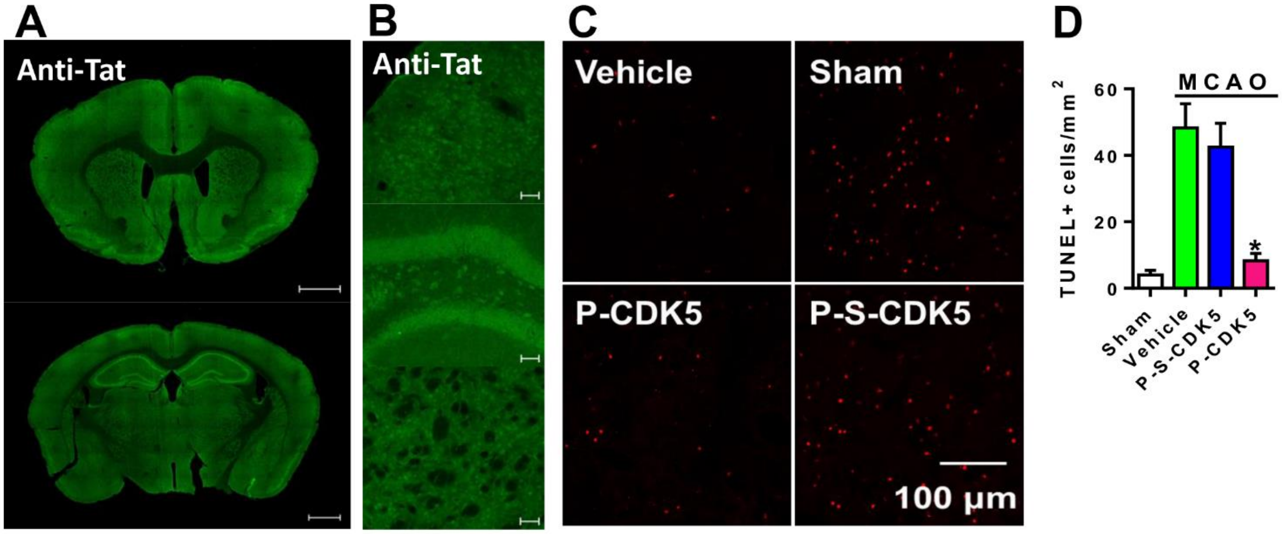

Supplement Figure 3. Tat-CDK5-CTM protects against stroke in vivo. (A) Representative images of immunofluorescence staining show the whole brain distribution of peptides. Bar=1mm. **(B)** Representative images of immunofluorescence staining show the distribution of peptides in cortex, DG and striatum. Bar=100μm. **(C)** Representative images show the TUNEL-labeled cells in the striatum of mice. Scale bar=100um. **(D)** Bar graph shows the numbers of the TUNEL-labeled cells. Data are mean ± SEM (ANOVA, *P<0.05; n=6).

References

[1] Pei L, Shang Y, Jin H, Wang S, Wei N, Yan H, et al. (2014). Dapk1-p53 interaction converges necrotic and apoptotic pathways of ischemic neuronal death. The Journal of neuroscience. 34:6546-6556

[2] Tong H, Liu L, Jin Y, Du L, Yin Y, Qian Q, et al. (2012). Dwarf and low-tillering acts as a direct downstream target of a gsk3/shaggy-like kinase to mediate brassinosteroid responses in rice. The Plant cell. 24:2562-2577

[3] Tu W, Xu X, Peng L, Zhong X, Zhang W, Soundarapandian MM, et al. (2010). Dapk1 interaction with nmda receptor nr2b subunits mediates brain damage in stroke. Cell. 140:222-234

[4] Pei L, Wang S, Jin H, Bi L, Wei N, Yan H, et al. (2015). A novel mechanism of spine damages in stroke via dapk1 and tau. Cereb Cortex. 25:4559-4571

[5] Wang X, Pei L, Yan H, Wang Z, Wei N, Wang S, et al.(2014). Intervention of death-associated protein kinase 1-p53 interaction exerts the therapeutic effects against stroke. Stroke. 45:3089-3091

© 2018. Zhou YF et al. Published online at http://www.aginganddisease.org/EN/10.14336/AD.2018.1225
